# miR-152-3p Overcomes Temozolomide Resistance in Glioblastoma by Targeting TGF-α and Enhancing Apoptosis

**DOI:** 10.32604/or.2026.083962

**Published:** 2026-08-13

**Authors:** Chun-Nun Chao, Chiung-Yao Fang, Chia-Hsin Hou, Jen-Tsung Yang, Yu-Ping Wu, Jui-Chieh Chen

**Affiliations:** 1 Department of Pediatrics, Ditmanson Medical Foundation Chiayi Christian Hospital, Chiayi, Taiwan; 2 Institute of Molecular Biology, National Chung Cheng University, Chiayi, Taiwan; 3 Department of Medical Research, Ditmanson Medical Foundation Chiayi Christian Hospital, Chiayi, Taiwan; 4 Department of Neurosurgery, Chang Gung Memorial Hospital, Chiayi, Taiwan; 5 College of Medicine, Chang Gung University, Taoyuan, Taiwan; 6 Department of Biochemical Science and Technology, National Chiayi University, Chiayi, Taiwan

**Keywords:** Glioblastoma (GBM), temozolomide (TMZ), drug resistance, MiR-152-3p, transforming growth factor-alpha (TGF-α)

## Abstract

**Objectives:** Temozolomide (TMZ) resistance remains a major challenge in glioblastoma (GBM) treatment. This study investigated the role of miR-152-3p and its downstream target, transforming growth factor-α (TGF-α), in regulating TMZ sensitivity in GBM. **Methods:** Public GEO and CGGA datasets were analyzed to evaluate the expression and prognostic significance of miR-152-3p. TMZ-resistant GBM cell lines (U87MGR and DBTRG-05MGR) were established by continuous TMZ exposure. Gain- and loss-of-function experiments were performed using miR-152-3p mimics and inhibitors. Cell viability, apoptosis, and TGF-α expression were assessed by MTT, qRT-PCR, and Western blot analyses. **Results:** miR-152-3p expression was significantly decreased in recurrent GBM and was associated with poor overall survival. TMZ-resistant GBM cells exhibited lower miR-152-3p expression than parental cells. Bioinformatic analyses identified TGF-α as a potential target of miR-152-3p. Overexpression of miR-152-3p suppressed TGF-α expression, reduced cell viability, and enhanced TMZ-induced apoptosis in resistant GBM cells. TGF-α knockdown similarly restored TMZ sensitivity. Conversely, inhibition of miR-152-3p increased TGF-α expression and attenuated TMZ-induced apoptotic signaling in TMZ-sensitive M059K cells. **Conclusion:** Together, our findings demonstrate that the miR-152-3p/TGF-α axis plays a critical role in regulating TMZ sensitivity in GBM, and targeting this pathway may represent a therapeutically relevant signaling axis to overcome chemoresistance.

## 1 Introduction

The evolution of oncology has witnessed a profound shift in cancer treatment paradigms, progressing from conventional surgery and radiotherapy to molecularly targeted therapies and personalized medicine [1]. Despite these substantial therapeutic advances, drug resistance remains a major challenge in cancer treatment, often leading to therapeutic failure and tumor recurrence. Among therapy-resistant malignancies, glioblastoma (GBM) remains one of the most aggressive and treatment-refractory cancers due to its highly invasive nature, marked molecular heterogeneity, and poor therapeutic responsiveness.

According to the 2021 World Health Organization (WHO) classification of central nervous system tumors, GBM is classified as a grade 4 tumor and represents the most common and aggressive primary brain malignancy in adults. This updated classification integrates molecular features with histopathological criteria, enabling more accurate tumor characterization and diagnosis [2]. Despite advances in GBM treatment, patient outcomes remain dismal, with a median overall survival of less than 15 months [3]. The standard treatment for patients with GBM includes maximal safe tumor resection, followed by concurrent radiotherapy and adjuvant Temozolomide (TMZ) therapy. This treatment regimen significantly improves the median overall survival and 2-year survival rates of patients [4].

As a blood–brain barrier-permeable alkylating agent, TMZ exerts its cytotoxic effects primarily through the covalent attachment of methyl groups to the O^6^ position of guanine residues within the host genome. This specific chemical modification subsequently triggers mismatch repair activation and propagates genomic instability, which culminates in programmed cell death in tumor cells. The long-term clinical utility of this therapeutic regimen, however, is progressively undermined by the evolution of both intrinsic and acquired resistance, accompanied by the clonal expansion of tolerant tumor cell populations. Therefore, deciphering the intricate cellular pathways and regulatory networks that govern TMZ resistance is paramount to mitigating therapeutic failure and identifying novel therapeutic strategies. Ultimately, uncovering these molecular drivers may facilitate the development of precision medicine approaches tailored to the unique genetic landscapes of individual GBM tumors [5].

MicroRNAs (miRNAs) are small endogenous regulatory RNAs that function as important post-transcriptional modulators of gene expression. Through sequence-specific interactions with complementary regions of target messenger RNAs (mRNAs), miRNAs regulate mRNA stability and translational activity, thereby influencing a wide range of cellular processes, including proliferation, invasion, and apoptosis [6]. Dysregulation of miRNA expression has been increasingly implicated in GBM pathogenesis and malignant progression. Among these dysregulated miRNAs, miR-152 has been reported to be significantly downregulated in GBM tissues compared with normal brain tissues, suggesting its potential role as a tumor suppressive regulator. Loss of miR-152 expression may contribute to enhanced proliferative and invasive capacities, as well as resistance to apoptosis in GBM cells. Therefore, restoration of miR-152 expression may represent a promising therapeutic strategy for improving treatment efficacy in GBM [7,8].

The potential of miRNAs to modulate cancer drug responsiveness through the regulation of resistance-associated signaling networks has been widely recognized [9]. However, the precise role of miR-152-3p in the development of TMZ resistance in GBM remains largely unclear. To address this knowledge gap, the present study investigated the functional significance and underlying molecular mechanisms of miR-152-3p in TMZ-resistant GBM. Specifically, we examined its clinical relevance and diagnostic potential, evaluated its effects on glioma cell survival under TMZ treatment, and determined whether transforming growth factor-α (TGF-α) serves as a critical downstream effector mediating TMZ resistance [10,11]. Collectively, these findings provide mechanistic insights into miR-152-3p-mediated regulation of TMZ resistance and may facilitate the development of novel therapeutic strategies for overcoming chemoresistance in GBM.

## 2 Materials and Methods

### 2.1 Bioinformatic Analysis of Public Datasets

To investigate the expression patterns and clinical significance of miR-152 in GBM, we utilized two independent public databases: the Gene Expression Omnibus (GEO) and the Chinese Glioma Genome Atlas (CGGA). For the GEO dataset (GSE32466), comprising 12 paired samples of primary and recurrent GBM and generated using the Agilent-021827 Human miRNA Microarray V3 platform (GPL10850; miRBase release 12.0), pre-extracted gProcessedSignal values were used for subsequent analysis. These values represent the background-subtracted signal intensities generated by the Agilent Feature Extraction software, ensuring high data fidelity for Agilent miRNA microarray platforms. For the CGGA cohort, data were retrieved from the microRNA_array_198 dataset (data type: microRNA microarray; platform: Human v2.0 miRNA Expression BeadChip, Illumina) via the CGGA portal (http://www.cgga.org.cn/). The downloaded expression data had undergone quantile normalization using the standard preprocessing pipeline provided by the CGGA portal. The cohort initially consisted of 91 patients, including primary GBM (*n* = 81), secondary GBM (*n* = 6), and recurrent GBM (*n* = 4). The inclusion criteria for this study were: (i) histologically confirmed glioma (WHO grades II–IV), (ii) availability of miR-152 expression levels, and (iii) complete follow-up survival information. Overall survival (OS) was defined as the time interval from the date of initial histological diagnosis to the date of death or the last follow-up. Patients who were alive at the final follow-up or lost to follow-up were censored. Samples with missing OS data or ambiguous clinical status were excluded from the prognostic analysis to ensure the integrity of the findings. Samples with missing OS data (*n* = 10) or ambiguous clinical status were excluded from the prognostic analysis to ensure the integrity of the findings, resulting in a final analytical cohort of 81 primary glioma patients for survival evaluation. To address potential selection bias, subtype stratification was performed according to WHO grades and histological classifications, as detailed in Supplementary Table S1. Additionally, due to the lack of uniform records regarding prior treatments (e.g., extent of surgical resection or specific chemoradiotherapy regimens) in the public dataset, these factors were not included in the stratification, which is acknowledged as a limitation of the study. Detailed clinico-pathological characteristics of the CGGA cohort are summarized in Supplementary Table S1. For Cox regression analysis, an independent CGGA cohort containing 171 glioma patients with complete clinicopathological information was additionally analyzed (Supplementary Materials and Methods). Clinico-pathological covariates, including age, gender, WHO grade, and miR-152 expression levels, were evaluated. Variables showing statistical significance (*p* < 0.05) in the univariate Cox regression analysis were subsequently included in the multivariate Cox proportional hazards model to determine independent prognostic indicators. All statistical and bioinformatic analyses were performed using IBM SPSS Statistics 20 (IBM Corp., Armonk, NY, USA) and GraphPad Prism 10 (GraphPad Software, San Diego, CA, USA). A two-tailed *p*-value < 0.05 was considered statistically significant. Given that the subgroup stratifications and regression covariates were restricted to a limited number of pre-specified clinically relevant variables, no formal multiple testing corrections (such as Bonferroni correction) were applied.

### 2.2 Cell Lines and Cell Culture

Three human GBM cell lines—U87MG (Cat. No. HTB-14, GBM of unknown origin), DBTRG-05MG (Cat. No. CRL-2020), and M059K (Cat. No. CRL-2365)—and the human embryonic kidney cell line HEK293T (Cat. No. CRL-3216) were used in this study, all obtained from the American Type Culture Collection (ATCC; Manassas, VA, USA), which provided certification that the cell lines were free of mycoplasma contamination and verified their identity through short tandem repeat (STR) DNA profiling. Cells were cultured in Dulbecco’s Modified Eagle Medium (DMEM; Gibco; Thermo Fisher Scientific, Inc., Waltham, MA, USA; Cat. No. 12800082) supplemented with 10% fetal bovine serum (Gibco; Thermo Fisher Scientific, Inc.; Cat. No. A5256701) and 1% Penicillin-Streptomycin (containing 100 U/mL penicillin, and 100 μg/mL streptomycin (Gibco, Thermo Fisher Scientific, Inc.; Cat. No. 15140122)). All cells were maintained in a humidified incubator at 37°C with 5% CO_2_ and 95% air. To ensure experimental reliability and minimize the risk of cumulative contamination, all experiments were conducted using cells within a low passage number (less than 15 passages) after thawing from the original ATCC-certified stocks. The morphological integrity and growth kinetics were strictly monitored via phase-contrast microscopy (Model MIBH103; Hamlet Optics, Hwatang Photoelectric Co., Ltd., Taipei, Taiwan) to ensure the consistency of the cell models. TMZ was purchased from Sigma-Aldrich (Merck KGaA, Darmstadt, Germany; Cat. No. T2577). The TMZ was dissolved in dimethyl sulfoxide (DMSO) to prepare a 50 mM stock solution, filtered, and stored at −20°C protected from light. The stock was subsequently diluted with fresh culture medium to the desired concentrations immediately prior to each experiment, ensuring that the final DMSO concentration did not exceed 0.05%. TMZ-resistant cell lines, U87MGR and DBTRG-05MGR, were derived from their respective parental cell lines, U87MG and DBTRG-05MG. Cells were continuously exposed to gradually increasing concentrations of TMZ, with the final concentration reaching 250 μM, to establish a stable drug-resistant phenotype.

### 2.3 Establishment of TMZ-Resistant Cell Lines

TMZ-resistant derivatives (U87MGR and DBTRG-05MGR) were established by continuous exposure of parental cells to stepwise increasing concentrations of TMZ, starting from 15.625 μM and gradually escalating to 250 μM via intermediate concentrations of 31.25, 62.5, and 125 μM over a period exceeding six months. The TMZ concentration was increased every 2–3 weeks once cells resumed stable proliferation at each concentration. The resulting resistant cells were maintained in medium containing 250 μM TMZ. To eliminate acute drug effects, resistant cells were cultured in TMZ-free medium for at least 72 h prior to all downstream experiments. For IC50 determination, parental and resistant cells were seeded into 96-well plates at a density of 5 × 10^3^ cells/well and exposed to various concentrations of TMZ (0, 15.625, 31.25, 62.5, 125, 250, 500, and 1000 μM) for 48 h. Subsequently, cell viability was evaluated using the MTT assay. IC50 values were determined using GraphPad Prism 10 (GraphPad Software, San Diego, CA, USA) by fitting the log(inhibitor) versus normalized response curve with a variable slope.

### 2.4 Cell Viability Assay

Cell viability was evaluated using the MTT assay. Cells (5000 cells/well) were inoculated into 96-well microplates and maintained overnight for proper adherence. Following overnight attachment, a 4-h starvation period in serum-free DMEM was introduced for cell synchronization, after which TMZ treatments were initiated. Subsequently, cells were treated with indicated concentrations of TMZ (0, 125, 250, and 500 μM) for 48 h. Four hours before the end of the incubation period, MTT solution (Sigma-Aldrich; Merck KGaA, Darmstadt, Germany; Cat. No. M2128) was added to each well at a final concentration of 0.5 mg/mL to allow the formation of formazan crystals. Formazan crystals precipitated at the bottom were solubilized by adding 100 μL of DMSO per well after aspirated the supernatant. Optical density (OD) was quantified using a SpectraMax ABS Plus microplate reader (Molecular Devices, San Jose, CA, USA) at a detection wavelength of 550 nm, with reference corrections performed at 750 nm. Cell viability was normalized to the untreated control group. All experiments were conducted with at least three independent biological replicates, with each group containing three technical replicates to ensure reproducibility.

### 2.5 RNA Extraction and Quantitative Real-Time PCR (qRT-PCR)

Cellular total RNA was harvested using TRIzol reagent (Protech Technology Enterprise Co., Ltd., Taipei, Taiwan; Cat. No. PT-KP200CT) in strict accordance with the manufacturer’s guidelines. The expression levels of miR-152-3p were quantified by qRT-PCR. RNA concentration and purity were assessed spectrophotometrically, and samples with an A260/A280 ratio between 1.8 and 2.0 were used for subsequent analyses. For reverse transcription, the Mir-X™ miRNA First Strand Synthesis Kit (Cat. No. 638315; Takara Bio USA, Inc., Mountain View, CA, USA) was employed to convert total RNA into cDNA. The miR-152 specific forward primer is 5′-TCAGTGCATGACAGAACTTGG-3′, and the universal reverse primer (mRQ 3′ primer) supplied with the kit was used. The primer sequences for U6 small nuclear RNA (U6 snRNA) were as follows: 5′-CTCGCTTCGGCAGCACA-3′ (forward) and 5′-AACGCTTCACGAATTTGCGT-3′ (reverse). For TGF-α mRNA quantification, reverse transcription was performed using the PrimeScript™ RT Reagent Kit (Takara Bio, Inc., Shiga, Japan), and real-time PCR was carried out using the KAPA SYBR^®^ FAST qPCR Master Mix (Kapa Biosystems, Wilmington, MA, USA). The primer sequences used were as follows: TGF-α: 5′-TTGCTGCCACTCAGAAACAGTG-3′ (forward) and 5′-TTGATCTGCCACAGTCCACCTG-3′ (reverse); GAPDH: 5′-CACCCATGGCAAATTCCATGGCA-3′ (forward) and 5′-TCTAGACGGCAGGTCAGGTCCACC-3′ (reverse). All primer sequences were custom-synthesized by Genomics BioSci & Tech Co., Ltd. (New Taipei City, Taiwan). The qRT-PCR reactions were performed in a total volume of 20 μL per well. For miRNA quantification, the reaction mixture contained 10 μL of 2× SYBR Advantage qPCR Master Mix, 0.4 μL of miR-152-specific forward primer (10 μM), 0.4 μL of mRQ 3′ Universal Primer (10 μM), 2.0 μL of cDNA template, and 7.2 μL of sterile dH_2_O. For mRNA quantification, each 20 μL reaction mixture contained 10 μL of KAPA SYBR FAST qPCR Master Mix, 0.4 μL of forward primer (10 μM), 0.4 μL of reverse primer (10 μM), 2.0 μL of cDNA template, and 7.2 μL of sterile dH_2_O. All qRT-PCR reactions were conducted on the MyGo PCR Detection System (IT-IS Life Science Ltd., Dublin, Ireland) under conditions recommended by the respective kit manufacturers. Amplification was driven by a 40-cycle program, with each cycle consisting of a brief 95°C denaturation (5 s) followed by an annealing/extension phase at 60°C for 30 s. Melt curve analysis was performed to confirm amplification specificity. Fold changes in target gene expression were calculated using the comparative Ct (2^−ΔΔCT^) method, with expression levels normalized to U6 for miRNAs and GAPDH for mRNAs. All qRT-PCR experiments were performed with at least three independent biological replicates, with each sample analyzed in technical triplicate to ensure precision.

### 2.6 Western Blot

To extract total protein, cell pellets were subjected to lysis in RIPA buffer (50 mM Tris-HCl, pH 7.4, 150 mM NaCl, 1% NP-40, 0.5% sodium deoxycholate, and 0.1% SDS) complemented with inhibitor cocktails (Sigma-Aldrich; Merck KGaA, Darmstadt, Germany; Cat. No. P8340). The protein concentrations of the resulting supernatants were verified via the BCA method (Thermo Fisher Scientific Inc., Waltham, MA, USA; Cat. No. 23225). Denatured protein samples (30 μg/lane) underwent electrophoretic separation on SDS-PAGE gels (12%), followed by immobilization onto PVDF membranes. Nonspecific binding sites on the membrane were masked using a 5% non-fat milk solution for 1 h at room temperature (RT). The blots were then probed overnight at 4°C with primary antibodies, including anti-cleaved PARP1 (1:1000, Abcam, ab32064), which specifically recognizes the cleaved fragment of human PARP1; anti-cleaved caspase-3 (Asp175) (1:1000, Cell Signaling Technology, #9661), which detects only the cleaved fragments and does not cross-react with full-length caspase-3; anti-methylguanine-DNA methyltransferase (MGMT) (1:1000, LifeSpan BioSciences, Seattle, WA, USA; Cat. No. LS-C109440); anti-TGF-α (1:1000, Cell Signaling Technology, Danvers, MA, USA; Cat. No. #3715); and anti-α-tubulin (1:5000, Millipore, MAB1864) as a loading control. Unbound primary antibodies were removed by washing with Tris-buffered saline supplemented with 0.05% Tween 20, after which the membranes were incubated with appropriate HRP-linked secondary antibodies (goat anti-rabbit IgG, Cat. No. #AP132P; Millipore; 1:5000) for 1 h at RT. The thoroughly washed membranes were developed using chemiluminescent substrates (Thermo Fisher Scientific; Cat. No. 34580) and subsequently visualized on X-ray films for densitometric assessment. Densitometric analysis of Western blot bands was performed using UN-SCAN-IT gel 6.1 software (Silk Scientific, Inc., Orem, UT, USA), and protein expression levels were normalized to α-tubulin. Quantitative data were obtained from at least three independent biological replicates (*n* ≥ 3).

### 2.7 In Silico Prediction of Putative miR-152-3p Target Genes

Putative downstream targets of miR-152-3p were comprehensively screened using six online bioinformatic resources: miRCURY LNA miRNA Target Search (formerly miRSearch v3.0; https://www.qiagen.com), miRTarBase v6.0 (https://mirtarbase.cuhk.edu.cn/), miRDB (http://www.mirdb.org/), miRSystem (http://mirsystem.cgm.ntu.edu.tw/), miRmap (https://mirmap.ezlab.org), and TarBase v.8 (http://www.microrna.gr/tarbase). These databases integrate computational predictions with experimentally validated miRNA–target interactions. Candidate genes common to all six databases were identified by manual Venn diagram analysis and carried forward for subsequent validation. This high-stringency filtering strategy was designed to reduce false-positive predictions and strengthen confidence in target selection, while recognizing that certain bona fide targets might not meet the selection criteria and therefore could have been excluded.

### 2.8 miRNA Mimic and Inhibitor Transfection

Cells were seeded at a density of 2 × 10^5^ cells per well in 6-well plates. After overnight incubation, cells were transfected with 25 nM of either mirVana™ miR-152-3p miRNA mimic (5′-UCAGUGCAUGACAGAACUUGG-3′; Thermo Fisher Scientific, Cat. No. 4464060) or mirVana™ miRNA mimic negative control #1 (Mimic NC) (Thermo Fisher Scientific, Cat. No. 4464058). For each well, 50 pmol of miRNA oligonucleotides and 4 μL of TurboFect™ Transfection Reagent (Thermo Fisher Scientific, Cat. No. R0531) were separately diluted in serum-free medium, mixed gently, and incubated at RT for 20 min to allow complex formation before being added to the cells. The final culture volume was adjusted to 2 mL per well. For inhibition assays, cells were transfected with mirVana™ miR-152-3p miRNA inhibitor (5′-CCAAGUUCUGUCAUGCACUGA-3′; Thermo Fisher Scientific, Cat. No. 4464084) or mirVana™ miRNA inhibitor negative control #1 (Inhibitor NC) (Thermo Fisher Scientific, Cat. No. 4464079). After 72 h of transfection, cells were collected for RNA and protein extraction. Transfection efficiency was verified by qRT-PCR, showing increased miR-152-3p levels in mimic-transfected cells and decreased levels in inhibitor-transfected cells compared with their respective negative controls. The same cells were then used for MTT and Western blot analyses. Corresponding Mimic NC and Inhibitor NC oligonucleotides were transfected in parallel under identical experimental conditions to minimize nonspecific transfection-related effects. No obvious morphological alterations or excessive cell death were observed under the transfection conditions used in this study. All transfection experiments were performed with at least three independent biological replicates.

### 2.9 Silencing of TGF-α via Lentiviral Transduction

To achieve targeted depletion of TGF-α, recombinant lentiviral vectors expressing hairpins (shTGF-α#1: TRCN0000369237; shTGF-α#2: TRCN0000369238) and a corresponding non-targeting control vector (pLKO.1-shControl; TRC2.Scramble) were procured from the National RNAi Core Facility (Academia Sinica, Taipei, Taiwan). The respective hairpin oligonucleotides comprised the following sequences: shTGF-α#1, 5′-CCGGCCAGAAGAAGCAGGCCATCACCTCGAGGTGATGGCCTGCTTCTTCTGGTTTTTG-3′; shTGF-α#2, 5′-CCGGTGAAGGGAAGAACCGCTTGCTCTCGAGAGCAAGCGGTTCTTCCCTTCATTTTTG-3′; and shControl, 5′-CCGGCCTAAGGTTAAGTCGCCCTCGCTCGAGCGAGGGCGACTTAACCTTAGGTTTTT-3′. Generation of infectious viral particles was executed by transiently co-transfecting individual shRNA-expressing pLKO.1 plasmid (4 μg), the packaging component pCMV-ΔR8.91 (4 μg) and the envelope vector pMD.G (0.4 μg) into appropriate packaging cells. This lipid-mediated delivery was driven by TurboFect™ Transfection Reagent (Thermo Fisher Scientific, Cat. No. R0531) in strict accordance with the guidelines outlined by both the manufacturer and the sourcing repository. Conditioned viral supernatants were subsequently collected at 48 and 72 h post-transfection, cleared of cellular debris via passage through a 0.45-μm syringe filter, and stored at −80°C until needed. For transduction, GBM cells were plated in 6-well plates at a density of 2 × 10^5^ cells per well and allowed to establish monolayers overnight. Viral supernatants supplemented with 8 μg/mL polybrene were subsequently applied to the cells and maintained for 24 h to facilitate efficient viral uptake. At the end of the exposure interval, the culture medium was replenished with fresh complete medium, and the cells were maintained for an additional 48 h. To select for clones exhibits stable integration of TGF-α knockdown, the cultures were maintained under selective pressure using 5 μg/mL puromycin for 7 consecutive days, with a complete exchange of selective media performed every 2 days. Following the elimination period, the surviving populations were harvested, and the efficiency of TGF-α silencing was systematically cross-examined through both qRT-PCR and Western blot analysis. All transduction and knockdown experiments were independently performed with at least three biological replicates.

### 2.10 Statistical Analysis

Quantitative outcomes across all assays are formatted as mean ± standard error of the mean (SEM), pooling data acquired from a minimum of three independent experiments. For the analysis of public datasets, we utilized the built-in statistical engine of the CGGA portal and GraphPad Prism 10 (GraphPad Software, San Diego, CA, USA). Differential expression of miR-152 between two groups (e.g., Gender or GBM vs. rGBM) was evaluated using the unpaired Student’s *t*-test. When assessing numerical variations among multiple groups involving more than two categories (e.g., WHO grades II–IV or different histological subtypes), we executed a one-way analysis of variance (ANOVA) coupled with Tukey’s post-hoc test for multiple comparisons. Prognostic analysis was conducted using the Kaplan–Meier method to estimate overall survival (OS). Patients were stratified into high and low expression groups based on the median expression value of miR-152. The statistical significance of differences between survival curves was determined using the log-rank (Mantel–Cox) test. For all statistical tests, a *p*-value of less than 0.05 was considered to indicate a statistically significant difference.

## 3 Results

### 3.1 Downregulation of miR-152-3p in Recurrent GBM and Its Association with Poor Prognosis

**Figure 1:**
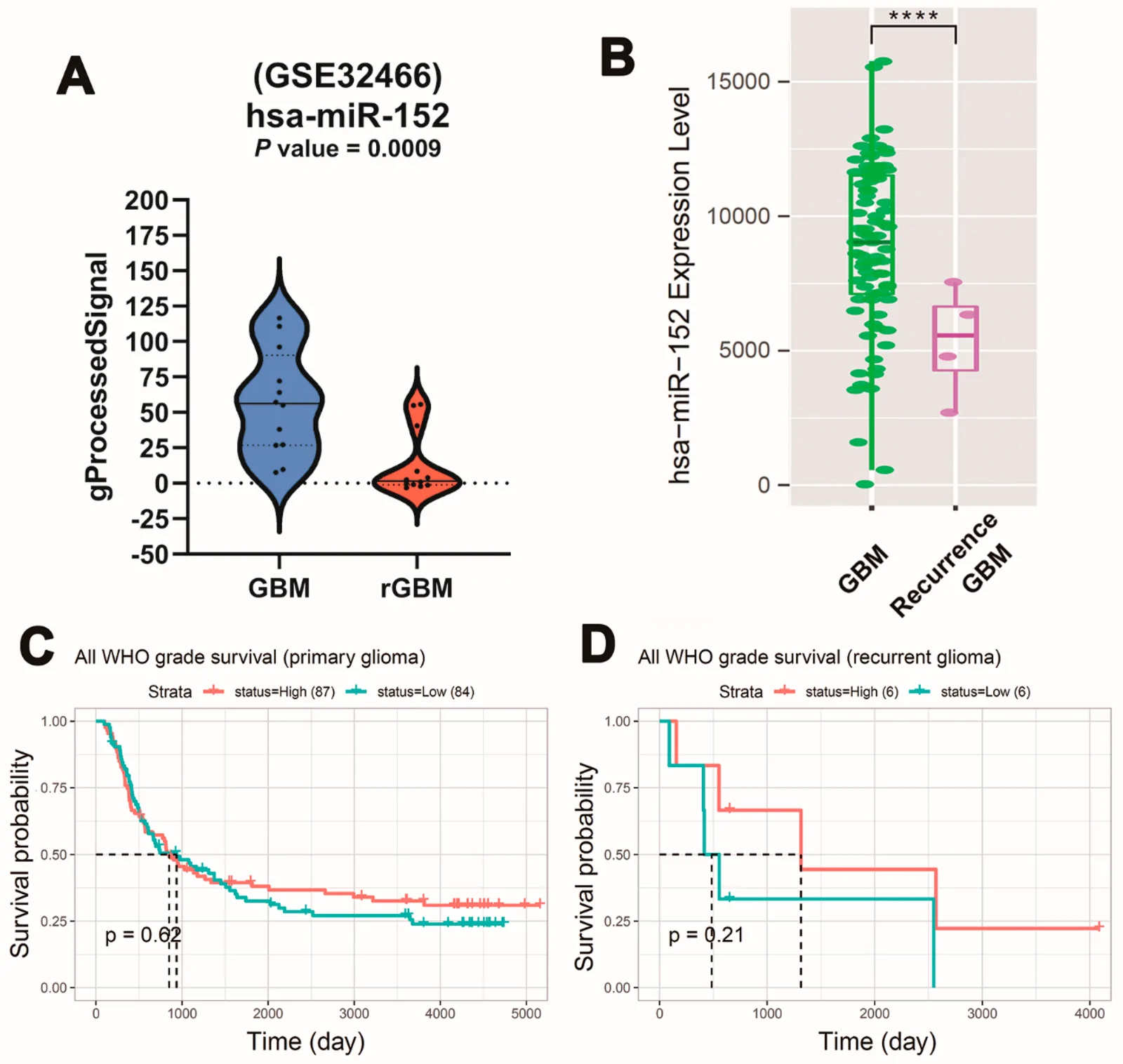
Expression and prognostic significance of miR-152 in glioma. (**A**) Violin plot showing the expression levels of miR-152 in primary GBM and rGBM in the GEO dataset (*p* = 0.0009). (**B**) Box plot illustrating miR-152 expression in GBM and rGBM based on the CGGA dataset (*p* = 3.5 × 10^−^^6^). (**C**) Kaplan–Meier survival analysis for patients with primary gliomas (all WHO grades) in the CGGA cohort stratified by high and low miR-152 expression (*p* = 0.62). (**D**) Kaplan–Meier survival analysis for patients with recurrent gliomas (all WHO grades) stratified by miR-152 expression (*p* = 0.21). ****p < 0.0001.

To elucidate the potential role of miR-152 in GBM progression, we analyzed its expression patterns in primary GBM and rGBM using two independent public datasets: the GEO and CGGA datasets. In the GEO dataset, miR-152 expression was significantly lower in the rGBM samples compared to the primary GBM samples (Fig. 1A). Analysis of the CGGA dataset also revealed a marked downregulation of miR-152 in rGBM relative to GBM (Fig. 1B).

To further investigate the prognostic significance of miR-152, patients from the CGGA cohort were stratified into high and low expression groups based on the median expression level. Kaplan–Meier survival analysis demonstrated that, among patients with primary gliomas of all WHO grades, low miR-152 expression was associated with a trend toward poorer overall survival, although the difference did not reach statistical significance (*p* = 0.62) (Fig. 1C). Similarly, in the recurrent glioma group, patients with low miR-152-3p expression exhibited a distinct trend toward worse overall survival compared to those in the high-expression group (Fig. 1D); however, this statistical significance was not achieved, likely due to the small sample size (*n* = 6 per group, *p* = 0.21). To further evaluate whether miR-152-3p serves as an independent prognostic biomarker, univariable and multivariable Cox proportional hazards regression analyses were performed using a subset of the CGGA cohort (*n* = 171) with complete clinicopathological data (Supplementary Table S2). Univariable analysis demonstrated that high miR-152-3p expression was significantly associated with favorable overall survival (HR = 0.612, 95% CI: 0.381–0.984, *p* = 0.043; Supplementary Table S3). Importantly, after adjustment for clinical covariates, including age, gender, and WHO grade, multivariable Cox regression analysis confirmed that miR-152-3p expression remained an independent prognostic factor in glioma patients (HR = 0.551, 95% CI: 0.313–0.969, *p* = 0.038; Supplementary Table S3). In contrast, higher WHO grade (III/IV vs. II) was identified as an independent risk factor for poor prognosis (HR = 2.964, 95% CI: 1.690–5.197, *p* < 0.001). These findings highlight the potential clinical utility of miR-152-3p as an independent prognostic indicator in glioma.

### 3.2 Downregulation of miR-152-3p in TMZ-Resistant GBM Cell Lines, and TGF-α as a Potential Target Gene

**Figure 2:**
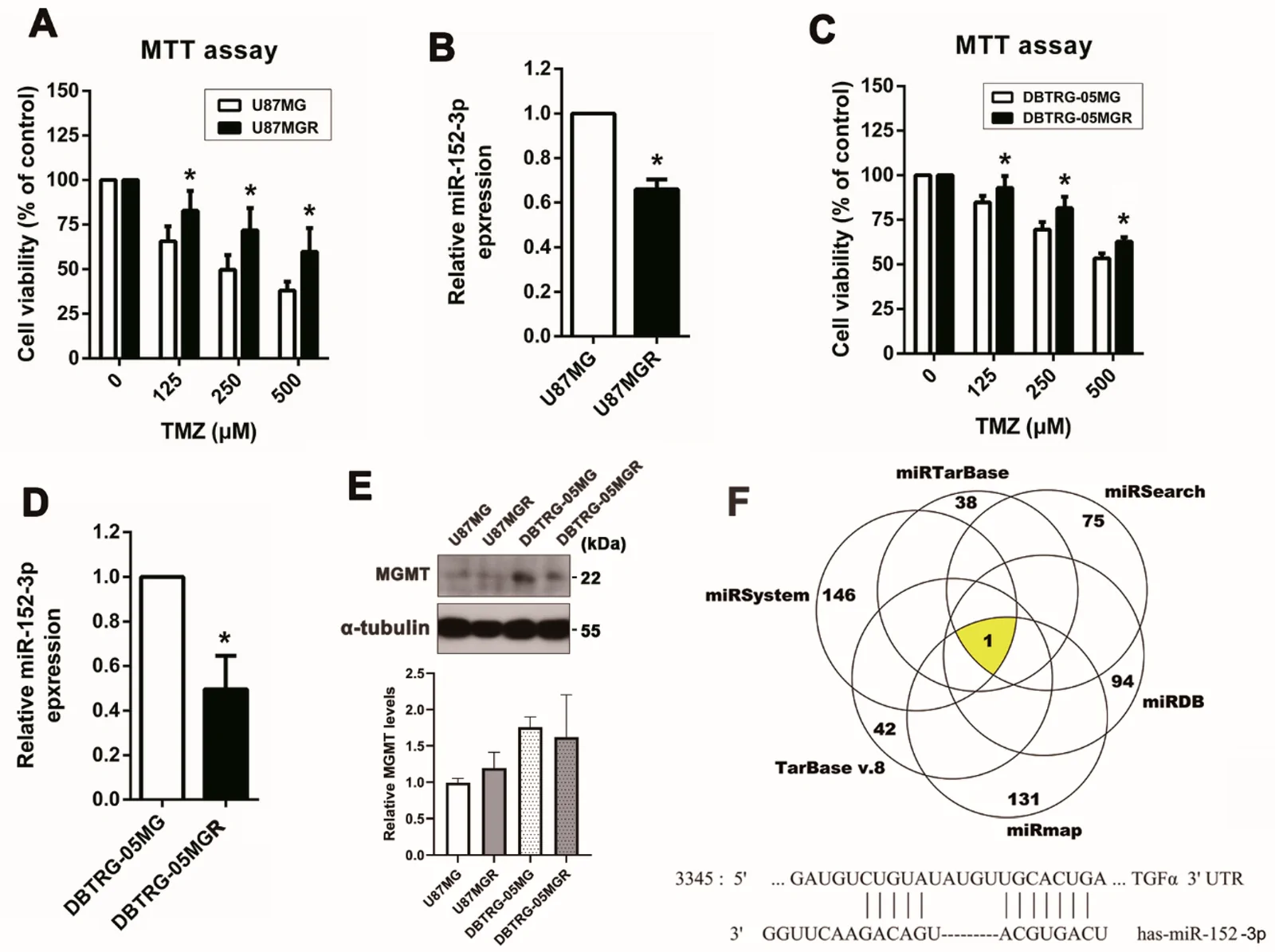
Expression of miR-152-3p in TMZ-resistant GBM cells and prediction of TGF-α as its potential target. (**A**) MTT assay was performed to evaluate the viability of U87MG cells and its TMZ-resistant derivative (U87MGR) following treatment with various concentrations of TMZ (0, 125, 250, and 500 μM). *p < 0.05 compared with parental U87MG cells. (**B**) qRT-PCR analysis of miR-152-3p expression in U87MGR cells, presented as relative expression compared with the parental U87MG cell line. *p < 0.05 compared with parental U87MG cells. (**C**) MTT assay was performed to evaluate the viability of DBTRG-05MG cells and its TMZ-resistant derivative (DBTRG-05MGR) following treatment with various concentrations of TMZ (0, 125, 250, and 500 μM). *p < 0.05 compared with parental DBTRG-05MG cells. (**D**) qRT-PCR analysis of miR-152-3p expression in DBTRG-05MGR cells, presented as relative expression compared with the parental DBTRG-05MG cell line. *p < 0.05 compared with parental DBTRG-05MG cells. (**E**) Western blot analysis of MGMT protein expression in U87MG, U87MGR, DBTRG-05MG, and DBTRG-05MGR cells. α-tubulin served as a loading control. (**F**) Bioinformatic prediction using six databases identified TGF-α as the only common predicted target of miR-152-3p, as shown in the yellow-highlighted intersection of the Venn diagram. The lower panel displays the predicted binding site between miR-152-3p and the 3′UTR of TGF-α mRNA.

To establish TMZ-resistant cell lines, U87MG and DBTRG-05MG cells were continuously exposed to gradually increasing concentrations of TMZ. TMZ-resistant derivatives, U87MGR and DBTRG-05MGR, were successfully generated. MTT assays revealed that U87MGR cells exhibited significantly higher cell viability under various TMZ concentrations compared to parental U87MG cells (Fig. 2A). Correspondingly, miR-152-3p expression was significantly decreased in U87MGR cells relative to parental U87MG cells (Fig. 2B). Similarly, DBTRG-05MGR cells exhibited significantly higher cell viability than parental DBTRG-05MG cells under various TMZ concentrations (Fig. 2C). A significant reduction in miR-152-3p expression was also observed in DBTRG-05MGR cells compared with parental DBTRG-05MG cells (Fig. 2D). In addition, increased IC50 values and resistance indices were observed in both resistant cell lines (Supplementary Table S4), further confirming the acquisition of TMZ resistance. These findings suggest that miR-152-3p may play a role in regulating TMZ resistance. To determine whether the TMZ-resistant phenotype was associated with the expression of **O^6^**-MGMT, Western blot analysis was performed on parental GBM cell lines and their TMZ-resistant derivatives. The results revealed that an increase in MGMT expression was not significant in the resistant cell lines compared with their parental counterparts (Fig. 2E), indicating that the development of TMZ resistance was not attributable to the upregulation of MGMT. Since miR-152-3p expression was markedly reduced in TMZ-resistant GBM cells, we next sought to identify its potential downstream target genes. Six online databases were employed for integrated bioinformatic analysis. A Venn diagram identified TGF-α as the only gene predicted by all six databases as a common target (Fig. 2F, up). Sequence alignment further demonstrated that miR-152-3p contains a complementary binding site within the 3′-untranslated region (3′UTR) of TGF-α (Fig. 2F, down), supporting it as a potential direct target of miR-152-3p.

### 3.3 miR-152-3p Mimic Suppresses TGF-α Expression and Enhances TMZ-Induced Apoptosis in U87MGR Cells

**Figure 3:**
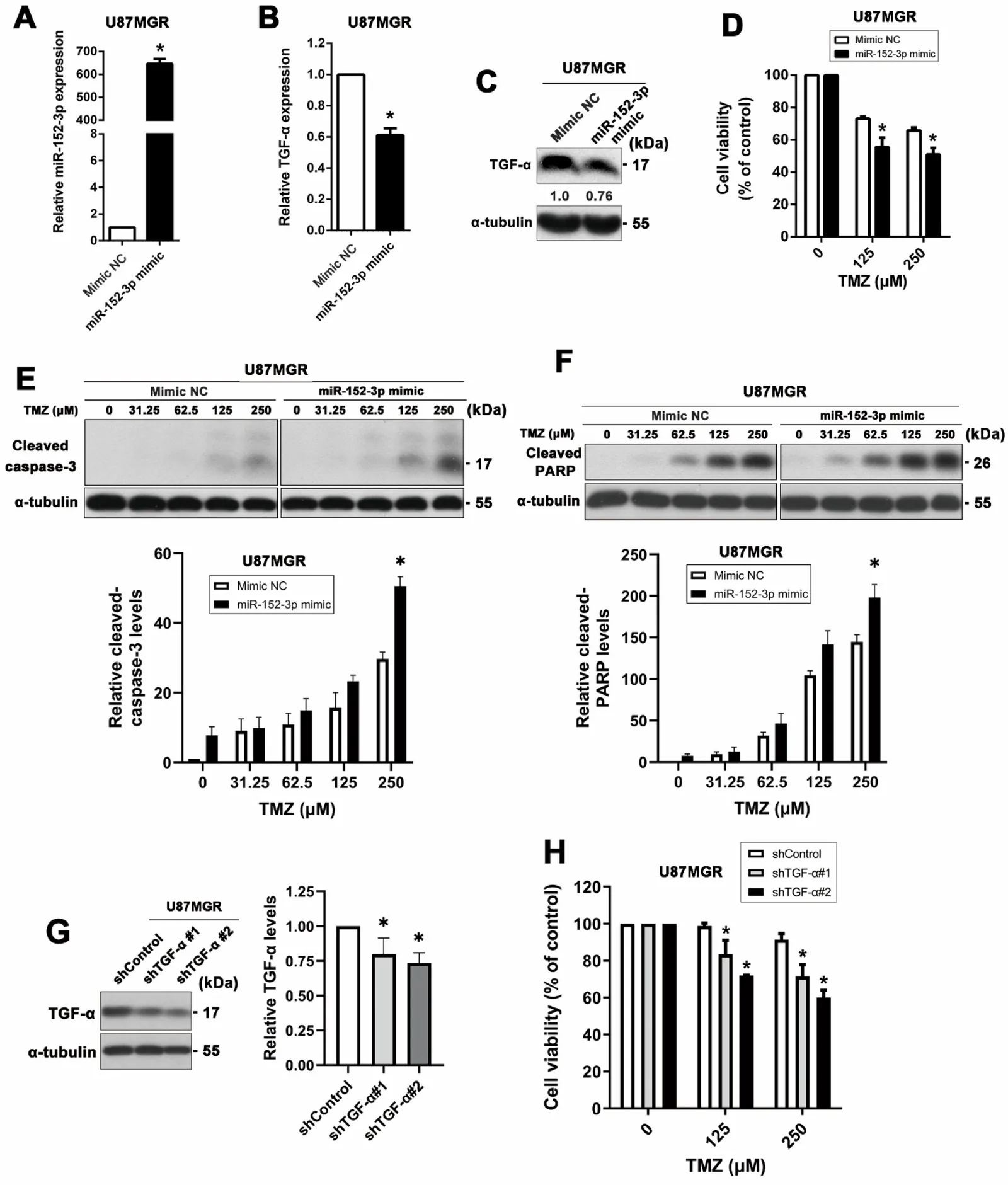
**miR-152-3p mimic suppresses TGF-α expression and enhances TMZ sensitivity in U87MGR cells.** (**A**–**C**) Cells were transfected with a miR-152-3p mimic or Mimic NC for 48 h. The expression levels of (A) miR-152-3p and (**B**) TGF-α mRNA were determined by qRT-PCR, whereas (**C**) TGF-α protein expression was assessed by Western blot. (**D**) Following transfection, cells were treated with various concentrations of TMZ for 48 h, and cell viability was measured using the MTT assay. (**E**) Representative Western blot images and densitometric quantification of cleaved caspase-3 (*n* = 3). The quantitative analysis confirmed that miR-152-3p mimic significantly increased the levels of cleaved caspase-3 in response to TMZ treatment. (**F**) Representative Western blot images and densitometric quantification of cleaved PARP (*n* = 3). The quantitative analysis confirmed that miR-152-3p mimic significantly increased the levels of cleaved PARP in response to TMZ treatment. (**G**) Cells were transduced with shControl or two shRNAs targeting TGF-α (shTGF-α #1 and shTGF-α #2), and TGF-α protein expression was confirmed by Western blot. (**H**) After TGF-α knockdown, cells were treated with increasing concentrations of TMZ for 48 h, and cell viability was evaluated using the MTT assay. *p < 0.05 compared with the Mimic NC or shControl group at the same TMZ concentration. α-Tubulin was used as the loading control.

In U87MGR cells, transfection with miR-152-3p mimic significantly increased the expression of miR-152-3p, as confirmed by qRT-PCR (Fig. 3A). This was accompanied by a reduction in TGF-α mRNA (Fig. 3B) and protein levels (Fig. 3C). Upon treatment with increasing concentrations of TMZ (125 and 250 μM), cell viability was significantly reduced in the miR-152-3p mimic group (Fig. 3D). Furthermore, the expression levels of cleaved caspase-3 and cleaved PARP increased in a dose-dependent manner, indicating that miR-152-3p mimic enhances TMZ-induced apoptosis (Fig. 3E,F). To further validate the role of TGF-α in TMZ resistance, TGF-α was knocked down in U87MGR cells, and the knockdown efficiency was confirmed by Western blot analysis (Fig. 3G). As shown in Fig. 3H, compared with shControl cells, TGF-α knockdown markedly reduced cell viability, indicating that silencing TGF-α restores the sensitivity of resistant GBM cells to TMZ.

### 3.4 miR-152-3p Mimic Reduces TGF-α Expression and Enhances TMZ Sensitivity in DBTRG-05MGR Cells

**Figure 4:**
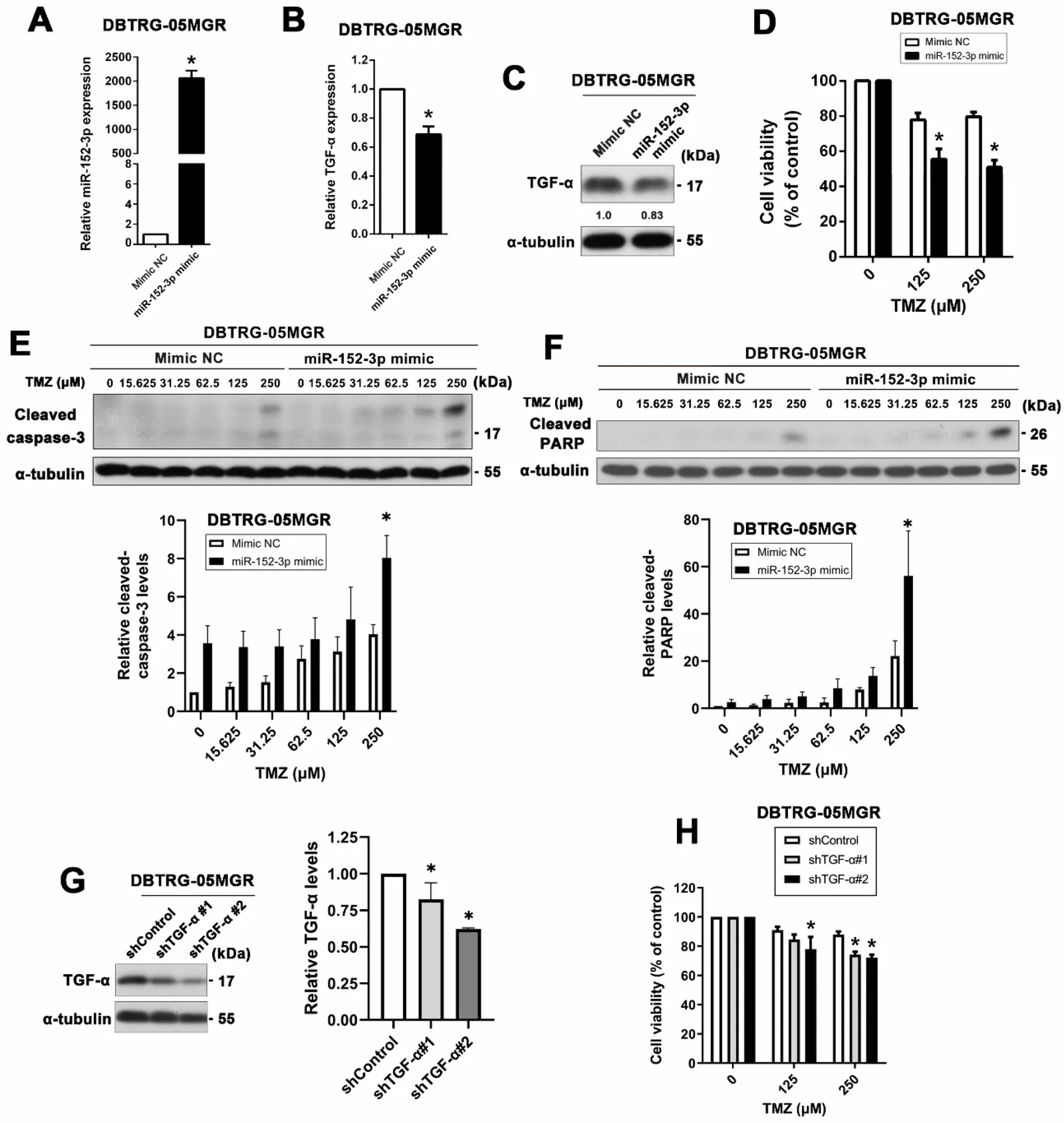
**miR-152-3p mimic reduces TGF-α expression and increases TMZ-induced apoptosis in DBTRG-05MGR cells.** (**A**) Cells were transfected with miR-152-3p mimic or Mimic NC for 48 h, and the expression levels of miR-152-3p were quantified by qRT-PCR. *p < 0.05 vs. Mimic NC. (**B**) Cells were transfected with miR-152-3p mimic or Mimic NC for 48 h, and the expression levels of TGF-α were quantified by qRT-PCR. *p < 0.05 vs. Mimic NC. (**C**) TGF-α protein expression was evaluated by Western blot. (**D**) Cell viability was measured using the MTT assay following 48 h of TMZ treatment at various concentrations. *p < 0.05 vs. Mimic NC. (**E**) Western blot analysis and corresponding densitometric quantification of cleaved caspase-3 (*n* = 4). The quantitative analysis indicated that miR-152-3p mimic significantly promoted TMZ-induced cleavage of caspase-3. *p < 0.05 compared with the Mimic NC group at the corresponding TMZ concentration. (**F**) Western blot analysis and corresponding densitometric quantification of cleaved PARP (*n* = 3). The quantitative analysis indicated that miR-152-3p mimic significantly promoted TMZ-induced cleavage of PARP. *p < 0.05 compared with the Mimic NC group at the corresponding TMZ concentration. (**G**) Cells were transduced with shControl or two independent shRNAs (shTGF-α #1 and shTGF-α #2), and TGF-α protein expression was confirmed by Western blot. The bar graph shows the densitometric quantification of TGF-α protein levels. *p < 0.05 vs. shControl. (**H**) Following TGF-α knockdown, cells were exposed to increasing concentrations of TMZ for 48 h, and viability was determined by the MTT assay. α-Tubulin served as the internal loading control. *p < 0.05 compared with the Mimic NC or shControl group at the same TMZ concentration.

To further verify whether the regulatory effect of miR-152-3p on TGF-α and its impact on TMZ sensitivity is consistent across different GBM cell lines, the analysis was extended to another TMZ-resistant GBM cell line, DBTRG-05MGR. miR-152-3p overexpression markedly increased miR-152-3p levels and significantly reduced TGF-α expression at both the mRNA and protein levels (Fig. 4A–C). After TMZ treatment, miR-152-3p-overexpressing cells exhibited significantly lower viability than the control cells (Fig. 4D). Also, cleaved caspase-3 and cleaved PARP levels were markedly higher in miR-152-3p-overexpressing cells in a dose-dependent manner with TMZ treatment (Fig. 4E,F). Furthermore, TGF-α knockdown enhanced TMZ sensitivity (Fig. 4G,H). Collectively, these findings demonstrate that miR-152-3p suppresses TGF-α expression and augments TMZ-induced apoptosis in DBTRG-05MGR cells, consistent with the results observed in U87MGR cells.

### 3.5 miR-152-3p Inhibition Promotes TGF-α Expression and Induces TMZ Resistance in M059K Cells

**Figure 5:**
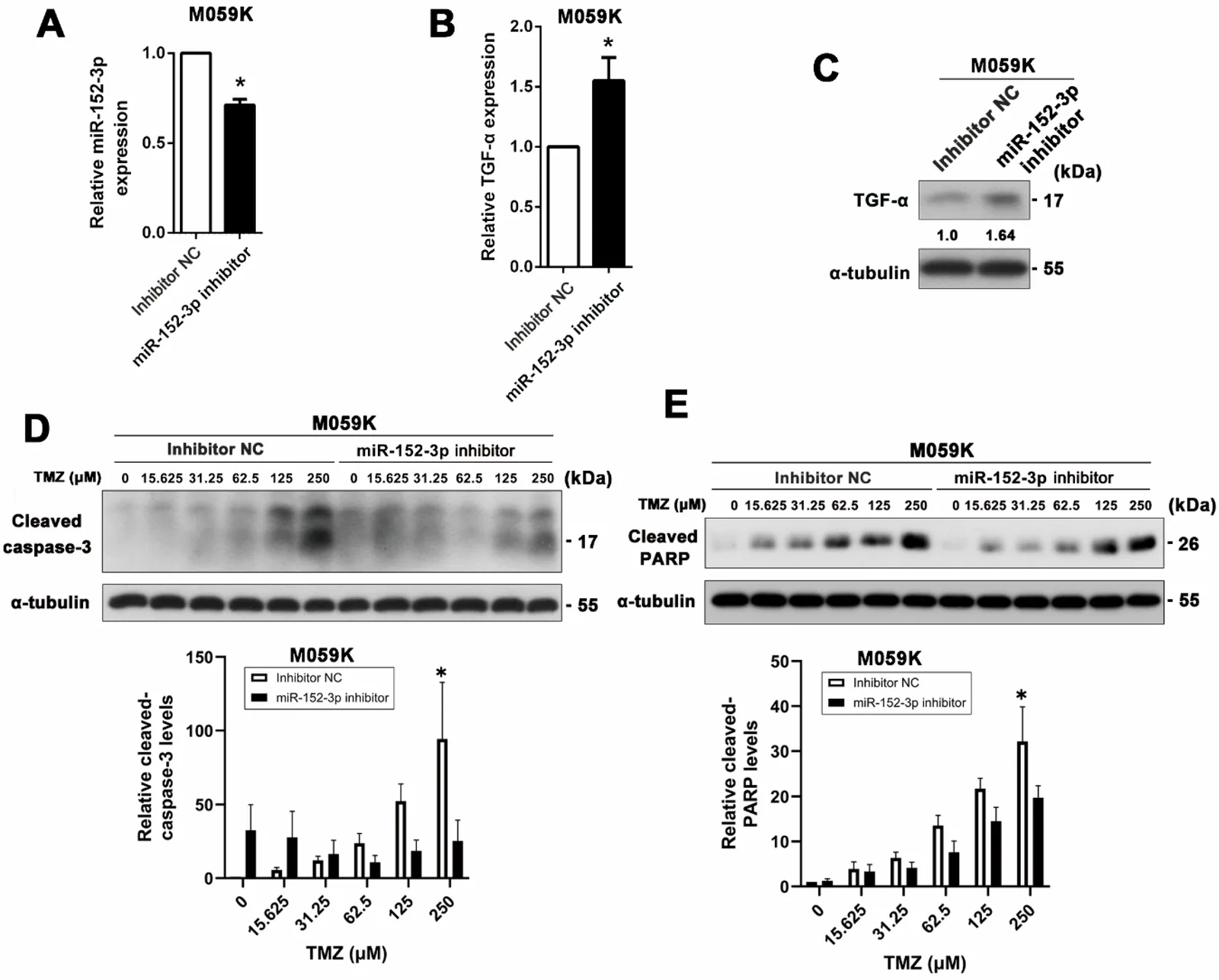
**Inhibition of miR-152-3p upregulates TGF-α expression and attenuates TMZ-induced apoptosis in M059K cells.** (**A**) qRT-PCR analysis showing reduced miR-152-3p expression upon miR-152-3p inhibitor transfection in M059K cells. *p < 0.05 vs. Inhibitor NC. (**B**) qRT-PCR analysis showing increased TGF-α mRNA levels upon miR-152-3p inhibitor transfection in M059K cells. *p < 0.05 vs. Inhibitor NC. (**C**) Western blot analysis confirming upregulation of TGF-α protein following miR-152-3p inhibition; α-tubulin was used as a loading control. (**D**) Western blot analysis and corresponding densitometric quantification of cleaved caspase-3 (*n* = 4). The quantitative analysis indicated that miR-152-3p inhibition significantly reduced TMZ-induced levels of cleaved caspase-3 compared with the Inhibitor NC group, particularly at higher TMZ concentrations. α-Tubulin served as a loading control. *p < 0.05 compared with Inhibitor NC. (**E**) Western blot analysis and corresponding densitometric quantification of cleaved PARP (*n* = 3). The quantitative analysis indicated that miR-152-3p inhibition significantly reduced TMZ-induced levels of cleaved PARP compared with the Inhibitor NC group, particularly at higher TMZ concentrations. α-Tubulin served as a loading control. *p < 0.05 compared with Inhibitor NC.

To further verify whether the miR-152-3p/TGF-α axis similarly regulates TMZ-induced apoptosis in TMZ-sensitive GBM cells, M059K cells—a relatively TMZ-sensitive line—were used as our research model. M059K exhibits a more pronounced drug response, making it suitable for investigating whether miR-152-3p inhibition would induce TMZ resistance. To investigate the function of miR-152-3p in TMZ-sensitive GBM cell lines, we further performed miR-152-3p inhibition experiments in M059K cells. qRT-PCR results showed that transfection with miR-152-3p inhibitor effectively downregulated miR-152-3p expression (Fig. 5A) and was accompanied by a significant increase in TGF-α mRNA and protein expression (Fig. 5B,C). Furthermore, Western blot analysis revealed that the expression levels of both cleaved caspase-3 and cleaved PARP were markedly reduced in the miR-152-3p inhibitor group following treatment with increasing concentrations of TMZ (Fig. 5D,E), suggesting that inhibition of miR-152-3p upregulates TGF-α expression and contributes to the development of TMZ resistance in sensitive M059K cells.

## 4 Discussion

Typically comprising approximately 22 nucleotides, miRNAs are a class of endogenous, non-coding RNA molecules characterized by their small size. These molecules play a crucial role in modulating gene expression by binding to complementary sequences within mRNA targets [6]. Previous studies have demonstrated that miR-152-3p is expressed at lower levels in GBM tissues compared to normal brain tissues [12]. Studies have also demonstrated that miR-152 plays a pivotal role in glioma cells, exerting a suppressive effect on cell proliferation and invasion while inducing apoptosis [7]. In addition, accumulating evidence suggests that restoration of miR-152 expression may enhance chemosensitivity and suppress malignant phenotypes in various cancers [12]. Hence, the utilization of miRNA-focused remedies is becoming a highly promising and innovative class of molecular therapeutic approaches for GBM treatment. Notably, miR-152 has been reported to effectively suppress the migratory and invasive capacities of prostate cancer cells by targeting TGF-α [13]. However, the correlation between miR-152 and TGF-α in GBM, as well as their impact on TMZ sensitivity, has not been elucidated. Thus, the present study investigated the relationship between miR-152 and its possible corresponding target, TGF-α, with a view to uncovering their contribution to TMZ resistance.

As key post-transcriptional regulators, miRNAs control the protein expression of many genes, thereby influencing a wide range of biological functions [14]. Studies have demonstrated that miRNAs within cancer cells can influence various cellular processes, including cell proliferation, migration, differentiation, cell cycle regulation, apoptosis, angiogenesis, and resistance to drugs [15,16]. Bioinformatics analysis demonstrated that miR-152-3p may be an upstream regulatory molecule of TGF-α, and its expression level is lower in relapsed patients with GBM. In TMZ-resistant GBM cells, the overexpression of miR-152-3p reduced the protein expression of TGF-α and restored its sensitivity to TMZ. Conversely, the inhibition of miR-152-3p can increase the protein expression of TGF-α and cause TMZ resistance in TMZ-sensitive GBM cells. Our findings align with prior investigations on the role of miR-152-3p in cisplatin resistance. Consistently, previous studies have suggested that restoration of miR-152 expression is associated with enhanced chemosensitivity and suppression of malignant progression in cancer cells [12]. Another study proved that the expression of miR-152 acts as a potent tumor suppressor in GBM stem cells, dramatically reducing cell proliferation, migration, and invasion while promoting apoptosis [17].

In the present study, both bioinformatic prediction and expression analyses consistently identified TGF-α as a potential downstream target of miR-152-3p. This finding is highly consistent with previous reports in prostate cancer. Zhu et al. demonstrated that miR-152 expression was negatively associated with pathological stage and Gleason score, and further confirmed TGF-α as a direct target of miR-152 using dual-luciferase reporter assays in PC-3 and DU145 cells. Moreover, a recent study by Tao et al. further revealed that circANKS1B promotes TGF-α expression through competitive sequestration of miR-152-3p. Collectively, these independent studies strengthen our observations and support the notion that the miR-152-3p/TGF-α axis plays an important role in prostate cancer progression, particularly in tumor invasion and migration. Nevertheless, additional experimental validation, including luciferase reporter assays in our experimental system, would further strengthen the direct regulatory relationship between miR-152-3p and TGF-α.

Recent studies have extensively elucidated the multifaceted tumor-suppressive functions of miR-152 family members in glioma pathogenesis, progression, and chemosensitivity. Specifically, miR-152-3p has been reported to be significantly downregulated in GBM tissues, where it exerts potent tumor-suppressive effects by inhibiting cell proliferation and invasion, promoting apoptosis, and reversing epigenetic dysregulation associated with DNA methylation pathways [12]. Beyond these classical suppressive mechanisms, miR-152-3p has been shown to modulate GBM stem cell properties via the XIST/miR-152/KLF4 axis [17]. Importantly, previous studies have further demonstrated that miR-152 is frequently silenced through promoter hypermethylation in multiple cancers, including glioma. This epigenetic inactivation, commonly mediated by DNMT1, contributes to the downregulation of miR-152-3p and may facilitate tumor progression, stemness maintenance, and therapeutic resistance [7,12]. While miR-152-3p regulates multiple targets, our findings, complemented by our most recent investigations, suggest that the TGF-α/EGFR axis serves as a primary functional driver of the chemoresistance phenotype observed in our model. Collectively, these findings highlight the tumor-suppressive role of miR-152 and suggest that restoring its expression may effectively inhibit glioma aggressiveness and improve the efficacy of chemotherapy.

As a ligand for the EGFR, TGF-α activates the EGFR signaling pathway following proteolytic cleavage and extracellular release of its transmembrane precursor protein [10,18]. Studies have shown that TGF-α promotes tumor cell growth and oncogene expression through autocrine or paracrine mechanisms, contributing to the initiation and progression of various cancers via processes such as cell proliferation, metastasis, drug resistance, and prognosis [19,20,21,22,23,24,25]. In the nervous system, TGF-α has been confirmed to regulate astrocyte development and function, with its signaling axis involving ErbB1 playing a crucial role in astrocyte maturation and proliferation; once this pathway is dysregulated, it can lead to abnormal astrocyte proliferation or dedifferentiation, thereby promoting glioma formation [26,27,28]. Particularly noteworthy, TGF-α has been reported to induce the transformation of differentiated mature astrocytes into stem-like cell types, providing a potential source of cancer stem cells, further impacting glioma invasiveness and treatment response [29]. Furthermore, multiple studies have identified a strong correlation between high TGF-α expression and disease recurrence as well as poor prognosis in various tumors. For instance, patients with high TGF-α expression in head and neck squamous cell carcinoma, esophageal cancer, and breast cancer exhibit significantly lower overall survival rates [22,30,31]. Although a large number of clinical samples are currently lacking to definitively clarify the exact role of TGF-α in patients with GBM, a comprehensive review of existing literature suggests that TGF-α has the potential to serve as a key biomarker for predicting GBM prognosis and drug resistance.

Regulatory mechanisms mediated by miR-152 appear to intersect with key oncogenic signaling pathways in glioma. For instance, the long non-coding RNA XIST has been reported to function as a competing endogenous RNA that sponges miR-152, thereby relieving repression of downstream oncogenic targets such as KLF4 and promoting glioma progression [8]. These lncRNA/miRNA/mRNA axes not only contribute to cell proliferation, glycolysis, and stemness in glioma cells but may also play a crucial role in therapeutic resistance. Notably, a study has shown that members of the miR-152 family are also involved in the proliferation and invasion of pituitary adenomas, exerting their suppressive effects by downregulating ALCAM expression [32]. These findings align with and expand upon our discovery that miR-152-3p sensitizes TMZ-resistant GBM cells by downregulating TGF-α, thereby enriching the current understanding of miR-152’s tumor-suppressive network. The convergent inhibition of multiple targets by miR-152, including TGF-α, FBXL7, DNMT1, and KLF4, underscores its central role in regulating tumor behavior and drug response. Therefore, modulating the miR-152 axis could serve not only as a potential prognostic biomarker but also as a potential therapeutic target to overcome TMZ resistance in GBM.

Although no upregulation of MGMT was detected in the TMZ-resistant cell lines we established (U87MGR and DBTRG-05MGR), prolonged TMZ exposure is known to promote chemoresistance through multiple MGMT-independent mechanisms, including mismatch repair deficiency, epigenetic reprogramming, and alterations in signaling pathways or noncoding RNA networks [33]. Based on our findings, we propose that downregulation of miR-152-3p in resistant GBM cells may lead to increased expression of TGF-α, which in turn activates downstream survival signaling (such as the EGFR/PI3K–AKT pathway), thereby contributing to reduced TMZ sensitivity [12]. To substantiate this proposed mechanism, our recent work has established both the necessity and sufficiency of TGF-α in driving TMZ resistance in GBM cells [10]. Specifically, elevated expression and secretion of TGF-α alone was sufficient to confer TMZ resistance and activate downstream p-EGFR (Tyr845) signaling in human GBM cells, while inhibition of TGF-α restored TMZ sensitivity *in vitro*. Furthermore, in subcutaneous xenograft models, TGF-α knockdown significantly suppressed tumor growth and enhanced TMZ sensitivity. In addition, analysis of clinical tissue arrays showed markedly elevated TGF-α protein levels in GBM tissues compared with astrocytoma and normal brain tissues. These complementary *in vitro*, *in vivo*, and clinical data strongly reinforce that the miR-152-3p/TGF-α axis identified in the present study represents a functionally relevant driver of TMZ resistance.

Beyond its therapeutic implications, our work should be situated within the broader landscape of computational and molecular approaches to brain cancer diagnosis and treatment. Identifying functionally validated molecular markers like the miR-152-3p/TGF-α axis is critical for linking molecular signatures to treatment response prediction. In this context, integrating such markers into multimodal diagnostic frameworks—such as the MDL-CA deep learning model, which employs a cross-attention mechanism to combine genomic and MRI data for accurate brain cancer diagnosis [34]—holds significant promise for advancing precision oncology in GBM. Furthermore, integrating *in silico* drug screening could enhance the translational relevance of our findings. Computational approaches may help identify small molecules that mimic miR-152-3p function or inhibit the TGF-α/EGFR signaling axis, thereby facilitating the development of novel therapeutic strategies.

Nevertheless, several limitations of this study should be acknowledged. The transcriptomic analyses were primarily based on publicly available bulk datasets, including GEO and CGGA, which may be affected by intratumoral heterogeneity, differences in cellular composition, and technical variations associated with sample processing and platform-specific normalization methods. These inherent limitations may introduce biological and technical biases that could influence the interpretation of expression patterns and survival associations. Therefore, the bioinformatic findings should be interpreted with caution [35]. Future studies incorporating single-cell transcriptomic analyses and larger prospective clinical cohorts may further clarify the clinical significance and cellular context of miR-152-3p in GBM progression and TMZ resistance. To the best of our knowledge, this is the first study to elucidate the mechanism by which miR-152-3p modulates TMZ sensitivity in GBM cells through direct regulation of TGF-α. This study employed a multi-level approach integrating public dataset analyses and functional assays to demonstrate that miR-152-3p is significantly downregulated in recurrent GBM tissues and TMZ-resistant cell lines, and that it modulates TMZ-induced apoptosis by targeting TGF-α. Database analyses further revealed that miR-152-3p expression inversely correlates with TGF-α levels and disease recurrence. Functionally, restoring miR-152-3p expression in TMZ-resistant cells reduced TGF-α levels and enhanced TMZ sensitivity, whereas miR-152-3p inhibition in TMZ-sensitive cells increased TGF-α expression and resistance. These findings highlight the pivotal role of the miR-152-3p/TGF-α axis in mediating TMZ responsiveness and suggest its potential as both a predictive biomarker and a therapeutic target for overcoming TMZ resistance in GBM.

## Data Availability

The data in the current study is available from the corresponding author upon reasonable requests.

## Supplementary Materials

The supplementary material is available online at https://www.techscience.com/doi/10.32604/or.2026.083962/s1.
